# The Relationship Between Blood Perfusion in the Lower Extremities and Heart Rate Variability at Different Positions

**DOI:** 10.3389/fphys.2021.656527

**Published:** 2021-08-13

**Authors:** Shuyong Jia, Qizhen Wang, Hongyan Li, Xiaojing Song, Shuyou Wang, Weibo Zhang, Guangjun Wang

**Affiliations:** ^1^Institute of Acupuncture and Moxibustion, China Academy of Chinese Medical Sciences, Beijing, China; ^2^Institute of Basic Research in Clinical Medicine, China Academy of Chinese Medical Sciences, Beijing, China

**Keywords:** laser doppler blood perfusion, electrocardiogram, RMSSD, refined composite multiscale entropy, refined composite multiscale fuzzy entropy, positions

## Abstract

Previous studies have explored the relationship between the complexity of local blood flow signals and heart rate variability (HRV) under different thermal stimulations. However, the relationship between the complexity of local blood flow signals and HRV in different positions is not clear. In this study, healthy participants were placed in different body positions. The bilateral blood flux and ECG were monitored, and refined composite multiscale entropy (RC MSE) and refined composite multiscale fuzzy entropy (RC MFE) were used to measure the complexity of the local blood flux. The sample entropy was calculated to evaluate the HRV complexity. The change of body position did not affect the time domain or frequency domain of HRV, but did reverse the blood flux laterality of the lower extremities. Furthermore, there was a negative correlation between the complexity of right-side blood flux and sample entropy of HRV when the participant was in the -10 degrees position. These results provide a new perspective of the relationship between skin blood flux signals and cardiac function.

## Introduction

Previous studies have shown that there is a correlation between the skin blood flux perfusion of the left and right sides of the body. Whether due to thermal (Kubo et al., 2011), physical (Guangjun et al., 2012), or laser (Wang et al., 2012) stimulation, an increase in the blood perfusion of the body part contralateral to the stimulation occurs, suggesting that the skin blood flux regulation of bilateral body parts is internally correlated under certain conditions. Further studies have shown that the skin blood flux distribution in the same parts of the body exhibits laterality, whether at the body surface (Wang et al., 2012) or in the viscera (Mezentseva and Pertsov, 2019). Preliminary studies have shown that this laterality can be used to quantitatively evaluate the microcirculation perfusion status of different age groups (Wang et al., 2017). When the digestive tract was stimulated by cold water load, the laterality changed (Wang et al., 2017). As skin blood flux regulation is controlled by the autonomic nervous system, we believe that the laterality of skin blood flux distribution may be a result of autonomic nerve regulation. This suggests that autonomic nerve function regulation may differ between the two sides of the body; the function is lateralized. Recent studies have shown that autonomic nerve function is lateralized under specific stimulation conditions (Aghababaei Ziarati et al., 2020), which provides direct evidence for the lateralization of skin blood flux regulation.

In contrast, there is more and more evidence that microcirculation can be used to assess vascular function at the systemic level (IJzerman et al., 2003; Holowatz et al., 2008), and there is a close relationship between heart and vessel functions (Debbabi et al., 2010; Pinter et al., 2012). A correlation between regional skin blood flux and heart rate variability (HRV) when the body surface was stimulated by different temperatures has been reported (Wang et al., 2018, 2019). However, there are no reports focusing on the laterality of blood perfusion from the perspective of complexity measurement. The purpose of this study is to analyze the relationship between blood perfusion and HRV in healthy participants in different body positions.

## Materials and Methods

### Inclusion and Exclusion Criteria

Participants were required to be 18–60 years of age and healthy (no history of any medical conditions). Patients receiving medication affecting the cardiovascular system or autonomic regulation were excluded from this study. The participants were asked to avoid coffee, tea, and alcohol for 24 h prior to the study.

### Participants and Positions

**Figure 1:**
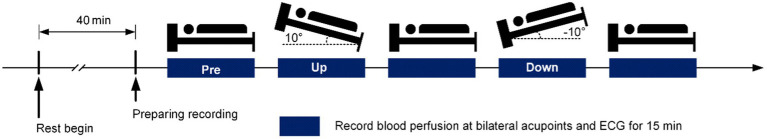
Schematic diagram of positions and recordings.

A total of 32 healthy participants were enrolled in current study, and 28 were included in the final statistical analysis. Each subject was paid for the participation. The characteristics of the participants are presented in Table 1. The study was conducted in a quiet, temperature-controlled (24–26°C) laboratory. Participants were instructed to lay flat on a multifunctional electric nursing bed (DB-3, Daermonda Medical Equipment Co., Ltd., Wuxi, China). As shown in Figure 1, the angle of the bed is adjustable. Following a 40-min period of cardiovascular stability, 15-min baseline ECG and bilateral Zusanli skin blood flux recordings were obtained with the participant in the horizontal position (Pre). The participant was then positioned at an angle of 10 degrees (up position) for 15 min before returning to a horizontal position. After a rest period of 15 min, the body position was changed to −10 degrees (down position) for 15 min before returning to horizontal. Both ECG and bilateral Zusanli skin blood flux were recorded at each body position. The changes in body position are shown in Figure 1.

**Table 1:** Participants’ characteristics.

n	Sex (female/male)	Age (years, mean ± SD)	Height (cm, mean ± SD)	Weight (kg, mean ± SD)
28	19/9	25.75 ± 2.35	166.54 ± 7.12	61.46 ± 10.97

### Measurement and Analysis of ECG Data

The participants maintained a supine position throughout the study. The ECG data was analyzed as previously described (Wang et al., 2013, 2015; Guangjun et al., 2014). Briefly, the ECG signals were recorded with standard II leads using the NeurOne system (NeurOne, MEGA electronics Ltd., Finland). The data were digitized at a sampling rate of 1,000 Hz. The raw data was exported in the EDF format and imported into Kubio HRV Premium software (Kubios Oy, Finland) for analysis (Niskanen et al., 2004). The length of analysis data was 15 min, and other analysis parameters were default. In the frequency domain, the power spectrum density was analyzed using the AR spectrum method in normalized units. Very low frequency (VLF) and low frequency (LF) were defined as 0–0.04 Hz and 0.04–0.15 Hz, respectively.

### Measurement and Analysis of Blood Perfusion Data

Both sides Zusanli acupoints (ST 36) were marked by senior acupuncture physicians. Blood perfusion signals were recorded using a PeriFlux System 5,000 (Perimed AB, Stockholm, Sweden) at a sample rate of 64 Hz and a time constant of 0.2 s. An optical fiber probe connected with a Periflux 5,000 was used to illuminate and collect the scattered light from the skin tissue. The probe was attached to the surface of interest with two-sided adhesive tape. The data were viewed using PeriSoft software for Windows (version 2.5.5, Perimed, Sweden), then exported in txt format and imported into MATLAB software (MathWorks, Natick, Massachusetts, United States) for analysis. The unit of blood perfusion is PU, which is the product of the number of moving blood cells in the measurement area and the average movement rate of blood cells. In consideration of sex-related differences in forearm skin microvascular reactivity (Stupin et al., 2019), male and female subjects were analyzed, respectively.

### Complexity of Blood Flux Signal

Refined composite multiscale entropy (RC MSE) and refined composite multiscale fuzzy entropy (RC MFE) were used to measure the complexity of the blood flux signal. The analytical methods of the MATLAB toolbox were used as previously described (Ahmed and Mandic, 2012; Azami and Escudero, 2017). A total of 15 min of blood flux data was used for the complexity analysis. The analysis parameters were *N* = 57,600 and scale = 20. The other parameters were default. A single index named complexity area index (CA) was calculated as the area under the multiscale entropy curve (Manor et al., 2010; Lu et al., 2012).

### Statistical Analysis

Data are presented as mean ± SE. Paired *t*-test was used to compare different positions and horizontal positions. The correlation between HRV and complexity of skin blood flow was calculated by Spearman’s correlation coefficient (SCC). All statistical analyses were conducted using MATLAB software. All reported *p* values are two-sided. Statistical significance was set at *p* < 0.05.

## Results

A total of 32 participants were recruited for this study; however, two participants did not complete the study and two had abnormal ECGs. The final analysis included 28 participants (Table 1).

### ECG Results

**Figure 2:**
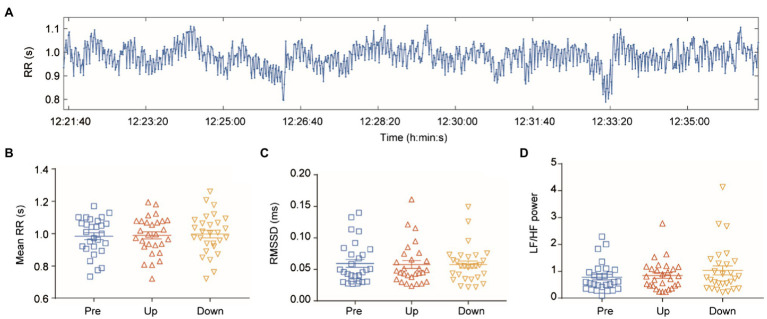
Change in heart rate variability (HRV). **(A)** RR intervals of the ECGs. **(B)** Mean RR intervals at each body position. **(C)** RMSSD at each body position. **(D)** LF/HF power ratio at each body position.

The RR interval signals are shown in Figure 2A. There was no significant difference in RR interval (Figure 2B; Supplementary Figures 1A, 2A), RMSSD (Figure 2C; Supplementary Figures 1B, 2B), and LF/HF ratio (Figure 2D; Supplementary Figures 1C, 2C) between the different body positions.

### Skin Blood Flux

**Figure 3:**
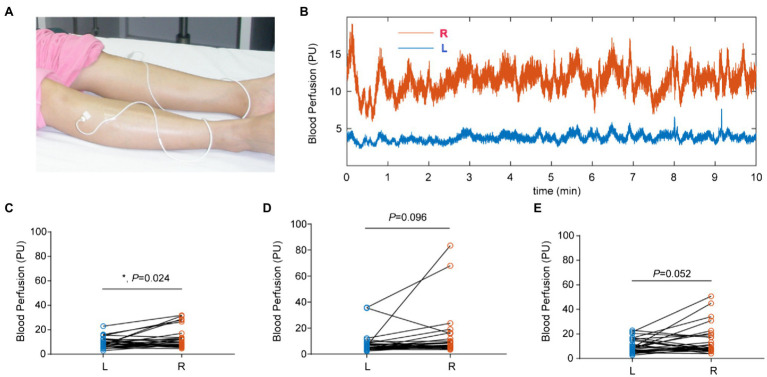
Blood perfusion in both lower extremities at different body positions. **(A)** Recording position at ST 36. **(B)** Raw data of blood flux in the left (blue) and right (red) lower extremities. **(C)** The blood flux was significantly different between the right (R) and left (L) sides in the horizontal position. **(D)** The blood flux was not significantly different between the R and L sides in the up position. **(E)** The blood flux was not significantly different between the R and L sides in the down position.

The average responses of blood perfusion to different body positions are shown in Figure 3. There was a significant difference in the skin blood flux between the left and right lower extremities in the horizontal position (Figure 3C); however, there was no difference in the skin blood flux between the left and right lower extremities in the up or down positions (Figures 3D,E). Subgroup analysis showed that regardless of female (Supplementary Figures 3A–C) or male (Supplementary Figures 4A–C), there was no significant difference in left and right blood perfusion.

### Relationship Between HRV and Blood Flux Signals

**Figure 4:**
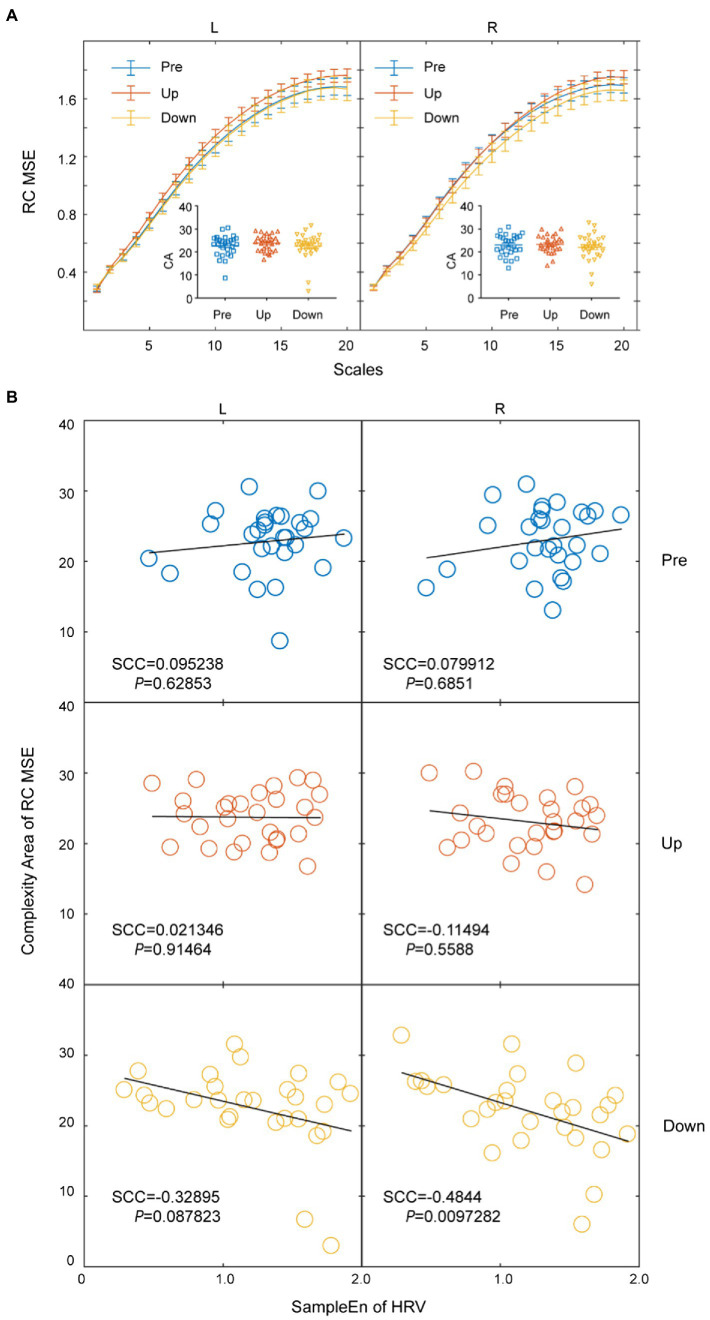
Relationship between sample entropy of HRV and refined composite multiscale entropy (RC MSE) of blood flux signals. **(A)**. There were no significant differences in the RC MSE of blood flux signals at different positions between the left and right lower extremities. **(B)**. The HRV is negatively correlated to the complexity area of the RC MSE blood flux signal in the right lower extremity in the down position, Spearman’s correlation coefficient (SCC).

**Figure 5:**
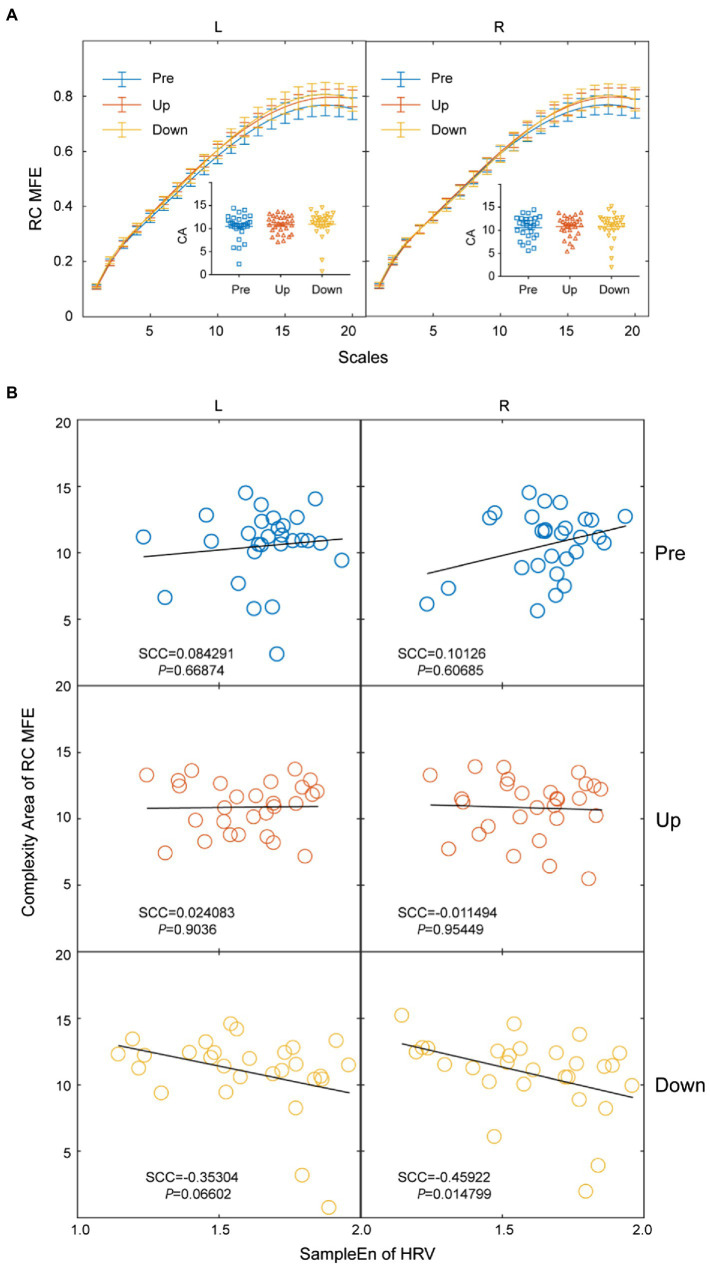
Relationship between sample entropy of HRV and refined composite multiscale fuzzy entropy (RC MFE) of blood flux signals **(A)** There were no significant differences in the RC MFE of blood flux signals at different positions between the left and right lower extremities **(B)** The HRV is negatively correlated to the complexity area of the RC MFE blood flux signal in the right lower extremity in the down position, SCC.

To discriminate the local blood flux pattern at different body positions, a time series of blood flux signals was analyzed using two methods of complexity analysis. There was no significant difference in local blood flux patterns between the different body positions on the RC MSE (Figure 4A) or RC MFE (Figure 5A) analyses in either lower extremity. However, the complexity of HRV was negatively correlated with the complexity of the right skin blood flux signal in the down position but not in other positions (Figures 4B, 5B).

## Discussion

This study found a negative correlation between the HRV and the right limb skin blood flux when the body was inclined at −10 degrees. However, there was no correlation between left blood flow and HRV in the same position. These results suggest that the regulation of bilateral blood flow is lateralized. Moreover, this correlation only occurred at the down position, indicating that certain conditions are required for the emergence of the laterality of the regulation of bilateral blood flow. These results are consistent with those of previous studies (Wang et al., 2018, 2019) that reported no correlation between HRV and skin blood flux under normal or near normal conditions, but a correlation between HRV and skin blood flux when the thermal stimulation reached a specific temperature.

The change of HRV in the up or down position is subtle and difficult to distinguish with traditional time domain or frequency domain analyses. Therefore, HRV is maintained within a normal range when the external stimulation does not exceed a certain threshold. However, different body positions can cause changes in tissue perfusion (Tapar et al., 2018). The results of this study suggest that the change of body position directly reversed the laterality of bilateral blood flow, indicating that although the heart and vascular system are a whole, they are relatively independent when controlled by the autonomic nervous system. In normal state or near normal state, there is no significant correlation between HRV and peripheral blood flow; this correlation is only observed when the external stimuli reach a certain threshold. The integration of the heart rate and vascular response indicate a correlation between HRV and peripheral blood flow in these conditions.

The regulation of the circulatory system is believed to be a nonlinear process (Liao et al., 2010); therefore, nonlinear dynamic analyses can provide information regarding the variability of skin blood flow oscillations (Liao et al., 2010; Jan et al., 2012). An MSE analysis provides a more powerful method for analyzing complexity measurements (Costa et al., 2002, 2005; Liao et al., 2020) of vascular dynamics. Heart rate signal is also believed to be a nonlinear process. The complexity of HR contains information beyond conventional time- and frequency-domain parameters, which could be sensitive to special conditions. It seems that some complexity parameters have higher regularity and predictability during environmental challenges (Young and Benton, 2015; Schneider et al., 2021). In current study, the intervention method is posture change, and the maximum tilt angle is 10 degrees. This method has little disturbance to the body, and it is more difficult to detect this change by conventional HRV related indicators. Therefore, The complexity of blood flow and heart rate signals is measured and the correlation between them is analyzed.

In this study, the RC MSE analysis results were less sensitive to the signal length than the RC MFE analysis results, suggesting that RC MSE should be used to assess the perfusion of the skin in different positions. Similarly, among irregular time series data in a wide range of time scales, the data with larger entropy are considered to be more complex (Kang et al., 2009). No correlations between the complexity of blood flow and HRV were observed in the horizontal or up positions in this study. A negative correlation between HRV and the complexity of the blood flow signal was observed in the right lower limb in the down position. These results highlight the laterality of the blood flow regulation.

Several studies have addressed the lateral distribution of blood flow (Wong et al., 1988; Yao et al., 2001; Ceylan et al., 2016). Significant differences in the distribution and regulation of skin blood flux in bilateral parts of the body have been reported (Wang et al., 2012; Mezentseva and Pertsov, 2019). Together, these studies confirm that the distribution of bilateral skin blood flux and its variation are asymmetric.

Generally, asymmetric or lateralization is considering as a fundamental characteristic in vertebrates (Vallortigara and Rogers, 2005) and invertebrate (Frasnelli, 2013). It is well known as hemispheric or cerebral laterality (Vallortigara and Rogers, 2005). Although, there is some evidence to support the existence of laterality in circulating blood flow signals (Wang et al., 2012; Mezentseva and Pertsov, 2019), this phenomenon is still not paid attention enough. We do not know whether this lateralization is an independent character or not associated with the handedness. This study seems to suggest that the laterality of microcirculation blood flow signal changes with the change of body position, especially in abnormal position. It means that laterality and that changes are closely related to systemic regulation. From the relevant perspective (Jia et al., 2020), the laterality of blood flow signal is closely related to autonomic nervous system, and the occurrence of some diseases of circulatory system, such as hypertension, is also the result of autonomic nervous dysfunction (Zubcevic et al., 2019; Yoo and Fu, 2020). Therefore, we speculate that the laterality of peripheral blood flow signals may be a window for understanding the regulation of autonomic nervous system.

Our research also has some shortcomings. The first limitation is that at each side, only one part blood flow was recorded. If blood flow of multiple parts on bilateral were recorded at the same time, the conclusion will be more representative and more reliable. The second disadvantage is that the blood flow and ECG were recorded only at two different positions. The third limitation is that the blood flow signal and HRV of a few subjects appear outlier data in abnormal posture, which indicates that the state of the subjects is not very well. The fourth deficiency is that there is a serious imbalance in the ratio of male to female, which leads to bias in the subgroup analysis of gender.

## Conclusion

Changes in body position can affect the correlation between skin blood flow and HRV.

## Data Availability Statement

The datasets presented in this study can be found in online repositories. The names of the repository/repositories and accession number(s) can be found at: https://doi.org/10.6084/m9.figshare.14907972.v1

## Ethics Statement

The studies involving human participants were reviewed and approved by Institutional Research Ethics Boards of Acupuncture & Moxibustion, China Academy of Chinese Medical Sciences. The patients/participants provided their written informed consent to participate in this study.

## Author Contributions

GW: conceptualization and funding acquisition. GW, SJ, and QW: methodology. GW and QW: software, formal analysis, and visualization. SJ, HL, XS, SW, WZ, and GW: resources. WZ and GW: data curation and supervision. SJ and GW: writing—original draft preparation, writing—review and editing, and project administration. All authors have read and agreed to the published version of the manuscript.

## Conflict of Interest

The authors declare that the research was conducted in the absence of any commercial or financial relationships that could be construed as a potential conflict of interest.

## Publisher’s Note

All claims expressed in this article are solely those of the authors and do not necessarily represent those of their affiliated organizations, or those of the publisher, the editors and the reviewers. Any product that may be evaluated in this article, or claim that may be made by its manufacturer, is not guaranteed or endorsed by the publisher.
