# Supplementary material for: The Relationship Between Blood Perfusion in the Lower Extremities and Heart Rate Variability at Different Positions
Source: Front Physiol. 2021 Aug 13;12:656527. doi: 10.3389/fphys.2021.656527 (PMC8414887; doi:10.3389/fphys.2021.656527)
Supplement: Supplementary file 1 [file Data_Sheet_1.docx]

The Relationship Between Blood Perfusion in the Lower Extremities and HRV at Different Positions

Shuyong Jia^1^*, Qizhen Wang^2^*, Hongyan Li^1^, Xiaojing Song^1^, Shuyong Wang^1^, Weibo Zhang^1^, Guangjun Wang^1^

1. Institute of Acupuncture and Moxibustion, China Academy of Chinese Medical Sciences, Beijing, China
2. Institute of Basic Research in Clinical Medicine, China Academy of Chinese Medical Sciences, Beijing, China

Shuyong Jia: shuyong6666@163.com

Qizhen Wang:18600759031@163.com

Hongyan Li: lhylhyz90@163.com

Xiaojing Song: xts2010@163.com

Shuyou Wang: wangsy15@126.com

Weibo Zhang: zhangweibo@hotmail.com

Guangjun Wang: tjuwgj@gmail.com

*: contributed equally

Corresponding Author: Guangjun Wang(tjuwgj@gmail.com)


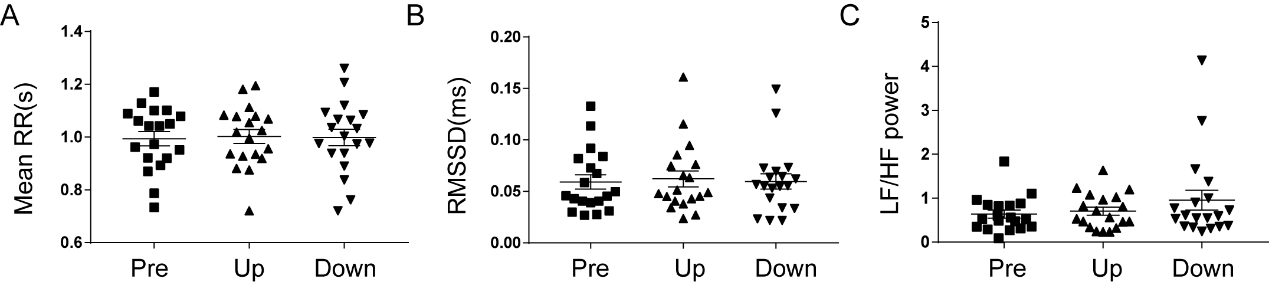


Supplementary Figure **1.** Change in heart rate variability of female subjects. (A) Mean RR intervals at each body position, *F*_(1.506,27.11)_=0.2095, *P*=0.749. (B) RMSSD at each body position, *F*_(1.492,26.85)_=0.5316, *P*=0.5421. (C) LF/HF power ratio at each body position, *F*_(1.08,19.44)_=1.974, *P*=0.1758.


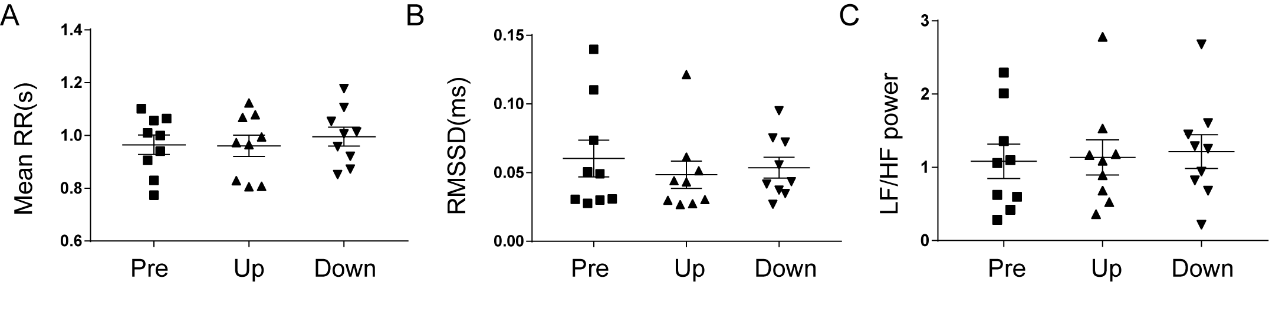
**Supplementary Figure 2.** Change in heart rate variability of male subjects. (A) Mean RR intervals at each body position, *F*_(1.415,11.32)_=1.428, *P*=0.2704. (B) RMSSD at each body position, *F*_(1.356,10.85)_=0.931, *P*=0.3866. (C) LF/HF power ratio at each body position, *F*_(1.266,10.13)_=0.5137, *P*=0.5319.


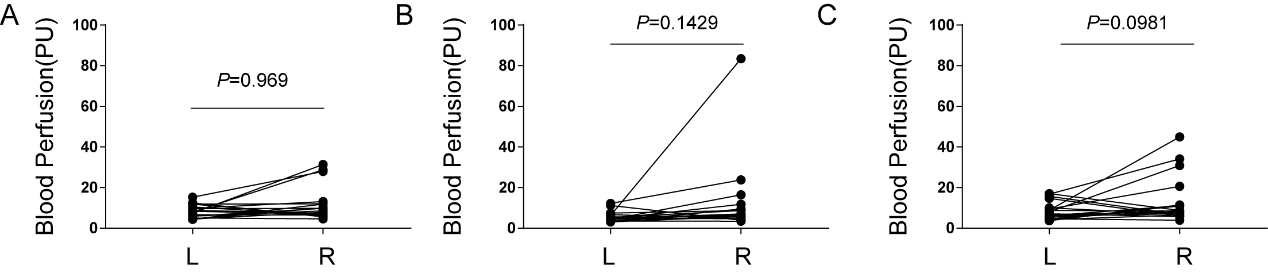


Supplementary Figure 3. Blood perfusion in both lower extremities at different body positions of female subjects. (A) The blood flux has no significant different between the right (R) and left (L) sides in the horizontal position. (B) Up position and (D) down position.


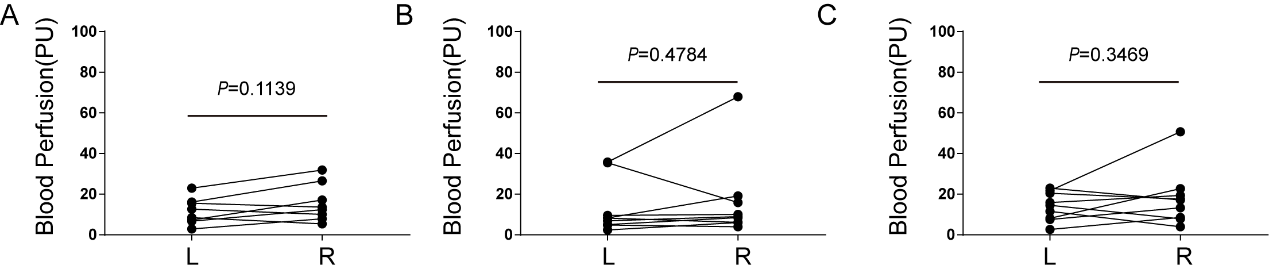


Supplementary Figure 4. Blood perfusion in both lower extremities at different body positions of male subjects. (A) The blood flux has no significant different between the right (R) and left (L) sides in the horizontal position. (B) Up position and (D) down position.
